# Neoadjuvant everolimus plus letrozole versus fluorouracil, epirubicin and cyclophosphamide for ER-positive, HER2-negative breast cancer: a randomized pilot trial

**DOI:** 10.1186/s12885-021-08612-y

**Published:** 2021-07-27

**Authors:** Wei Wu, Jiewen Chen, Heran Deng, Liang Jin, Zhanghai He, Nanyan Rao, Yan Nie, Yandan Yao, Yaping Yang, Fengxi Su, Jieqiong Liu

**Affiliations:** 1 Guangdong Provincial Key Laboratory of Malignant Tumor Epigenetics and Gene Regulation, Breast Tumor Center, Sun Yat-sen Memorial Hospital, Sun Yat-sen University, Yanjiang West Road 107#, Guangzhou, China; 2 Guangdong Provincial Key Laboratory of Malignant Tumor Epigenetics and Gene Regulation, Department of Pathology, Sun Yat-sen Memorial Hospital, Sun Yat-sen University, Guangzhou, China

**Keywords:** Randomized neoadjuvant pilot trial, Everolimus plus letrozole, Fluorouracil, Epirubicin and cyclophosphamide (FEC), ER-positive and HER2-negative breast cancer

## Abstract

**Background:**

Here we evaluated the feasibility, efficacy, tolerability, and treatment-mediated immune modulation of neoadjuvant everolimus plus letrozole versus chemotherapy in treating postmenopausal patients with ER-positive, HER2-negative breast cancer.

**Methods:**

Postmenopausal women with ER-positive, HER2-negative breast cancer who had a primary tumor > 2 cm or positive axillary lymph node(s) proofed by biopsy were randomly (1,1) enrolled to receive neoadjuvant everolimus plus letrozole for 18 weeks or fluorouracil, epirubicin plus cyclophosphamide (FEC) for 6 cycles before surgery. Primary outcome was feasibility of the trial. Secondary outcome included ultrasound response rate, pathological complete response rate, breast-conserving surgery rate, toxicities, treatment-mediated immune modulation and biomarkers.

**Results:**

Forty patients were randomized. Completion rate was 90.0% in the neoadjuvant endocrine therapy (NET) arm but 70.0% in the neoadjuvant chemotherapy (NAC) arm. The ultrasound response rate was 65.0% in NET arm and 40.0% in FEC arm, respectively. In terms of the adverse events, clearly favored NET arm. Everolimus plus letrozole increased the ratio of peripheral Tregs to CD4^+^ T cells and tumor PD-L1 expression, and decreased Ki67 index and tumor-infiltrating Tregs, and patients with a greater increase of tumor-specific CTLs showed more sensitive to NET.

**Conclusion:**

This pilot trial showed that neoadjuvant everolimus plus letrozole might achieve a favorable ultrasound response rate with low toxicities in treating postmenopausal ER-positive, HER2-negative breast cancer patients. Everolimus plus letrozole might have positive antitumoral immunity effects. Further large randomized controlled trials are needed to confirm our findings.

**Trail registration:**

A Trial of Neoadjuvant Everolimus Plus Letrozole Versus FEC in Women With ER-positive, HER2-negative Breast Cancer, registered on 07/04/2016 and first posted on 18/04/2016, NCT02742051.

**Supplementary Information:**

The online version contains supplementary material available at 10.1186/s12885-021-08612-y.

## Background

Neoadjuvant treatment either with chemotherapy or endocrine therapy is widely used for breast cancer patients [1]. Response to neoadjuvant chemotherapy (NAC) varies by estrogen receptor (ER) and human epidermal growth factor receptor 2 (HER2) statuses, with responses being lower in ER-positive, HER2-negative cancers as compared with HER2-overexpressing or triple-negative tumors as evidenced by significantly lower pathological complete response (pCR) rate and objective response rate (ORR) [2, 3]. ER-positive, HER2-negative breast cancer accounts for about 72.7% of all breast cancer cases [4], but it has a low ORR of less than 65% in response to NAC [2]. Neoadjuvant endocrine therapy (NET) is an important alternative to NAC for ER-positive, HER2-negative breast cancer, and previous trials established that NET with aromatase inhibitors (AIs) improved the response rates for postmenopausal women compared with tamoxifen [5, 6]. Nevertheless, a trial comparing letrozole NET with standard NAC (fluorouracil, epirubicin plus cyclophosphamide, FEC) in ER-positive postmenopausal breast cancer found that the ORR was 59.1% with letrozole, which was similar to that (54.5%) with chemotherapy [7]. Moreover, the recent NeoPAL trial which evaluate the efficacy of letrozole and cyclin-dependent kinase 4/6 inhibitors (CDKi) palbociclib versus chemotherapy as neoadjuvant therapy of high-risk ER-positive, HER2-negative breast cancer showed that letrozole combined with palbociclib was associated with poor pathological response of chemotherapy, and had a very similar ultrasound response rate with chemotherapy (55.5% vs. 53.3%) [8]. PALLET trial also demonstrated that ORR was not significantly different between palbociclib plus letrozole and letrozole monotherapy groups (*P* = 0.20; ORR: 54.3% vs. 49.5%) [9]. Thus, the need for finding other targeted drugs in combination with AIs that could sensitize ER-positive, HER2-negative breast tumors to endocrine therapy for this subgroup of patients is critical.

Activation of PI3K/Akt/mTOR pathway is a known mechanism of resistance to endocrine therapy [10–13]. A prior phase 2 trial showed that the response rate with 16 weeks of neoadjuvant mTOR inhibitor everolimus plus letrozole was significantly higher than that with letrozole plus placebo (68.1% vs. 59.1%) [14]. We hypothesized that for postmenopausal ER-positive, HER2-negative breast cancer, neoadjuvant everolimus plus letrozole could improve the response rate compared with neoadjuvant FEC chemotherapy. Additionally, mTOR pathway has immunoregulation effects. Several studies demonstrated that inhibition of mTOR may regulate immunosuppressive regulatory T cells (Tregs) in patients with metastatic renal cell carcinoma or prostate cancer [15–18]. No clinical studies have assessed the immunoregulatory effects of mTOR inhibitors in breast cancer patients so far.

Here we designed this open-label, randomized feasibility trial to evaluate the feasibility, efficacy and tolerability of neoadjuvant everolimus combined with letrozole versus neoadjuvant FEC chemotherapy in postmenopausal women with ER-positive, HER2-negative breast cancer. Due to the envisaged of recruitment challenges and uncertain intervention acceptability, this trial was conducted as a randomized phase II pilot trial. In addition, immunoregulatory effects of neoadjuvant therapy, and associations between blood immune cell subpopulations, tumor-specific cytotoxic T lymphocytes (CTLs) as well as tumor Ki67 index and tumor immune biomarkers, and response to NET or NAC were also assessed.

## Methods

### Study design, patients and randomization

**Fig. 1:**
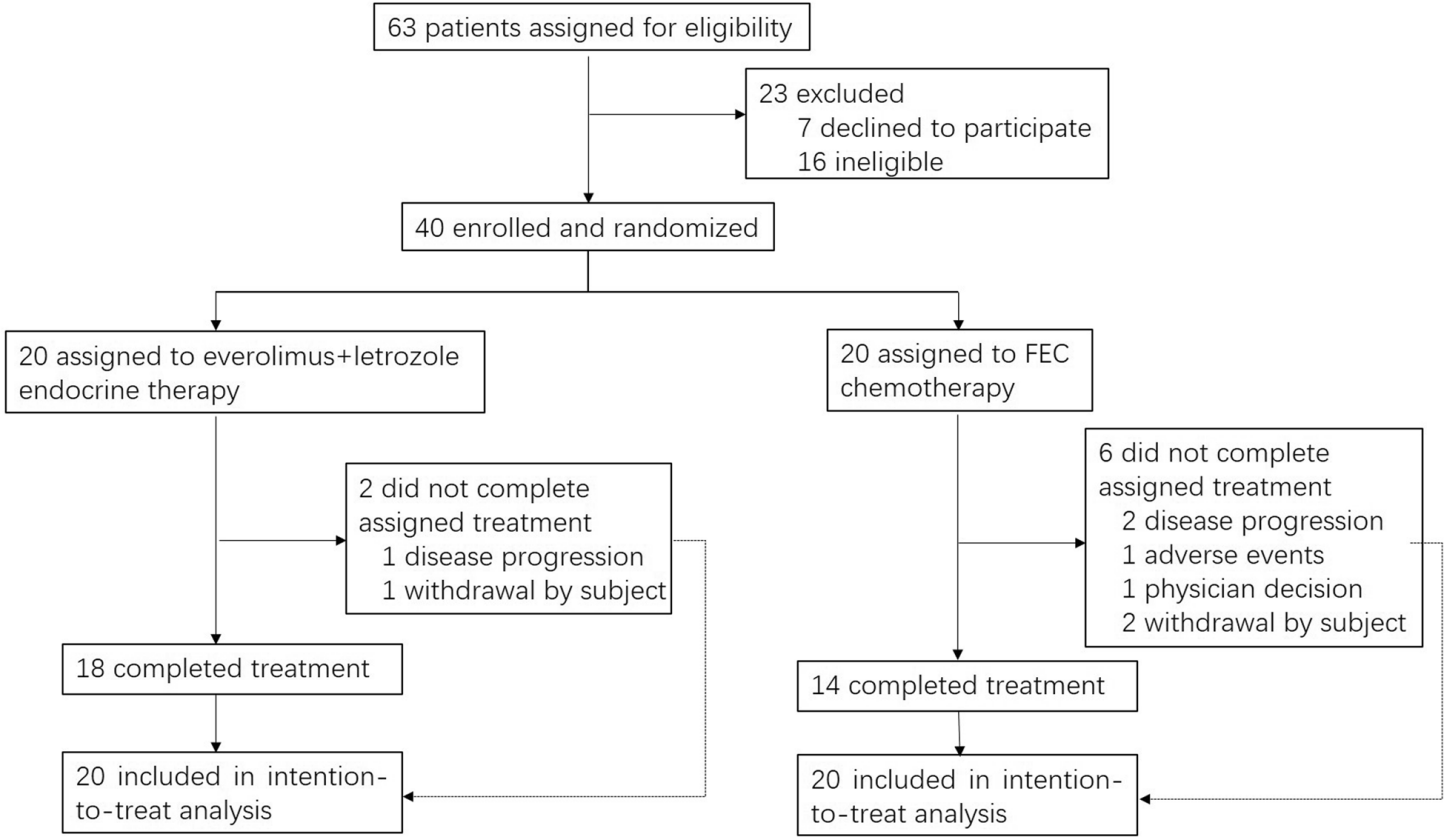
Study design flowchart

This phase II trial (NCT02742051) is a single-center, open, randomized pilot feasibility study. Forty postmenopausal women with stage M0, ER-positive, HER2-negative invasive breast cancer were randomly (1,1) enrolled to receive neoadjuvant everolimus plus letrozole for 18 weeks or neoadjuvant FEC for 6 cycles (21 days per cycle) before definitive surgery (Study design flowchart is shown in Fig. 1) at the Breast Tumor Center of the Sun Yat-sen Memorial Hospital. Random assignment was done with a computer-assisted randomization-allocation sequence with a block size of four. Key eligibility criteria included women aged less than 70 years old and presented with non-metastatic unilateral invasive ER-positive, HER2-negative breast cancer with a primary breast tumor > 2 cm by imaging and/or positive axillary lymph node(s) proofed by biopsy, and had no history of hormone therapy, chemotherapy, immunotherapy, breast cancer surgery and radiotherapy, and with an Eastern Cooperative Oncology Group performance status of 0–2. Key exclusion criteria were patients who had bilateral breast cancers or multifocal breast cancers or inflammatory breast cancers, had a history of prior treatment with mTOR inhibitors, and an allergic history or contraindication of any of the interventional drugs. Detailed eligibility and exclusion criteria were described in our previous trial protocol paper published in Trials [19].

This work had been reported in line with Consolidated Standards of Reporting Trials (CONSORT) Guidelines. This work was conducted in accordance with the principles of Good Clinical Practice, and the provisions of the Declaration of Helsinki.

### Procedures

A representative sample of core needle biopsies of the primary tumor was required for confirmation of ER/PR expression and lack of HER2 expression, as well as for other biomarker assessment including Ki67 index.

Patients were randomly (1,1, block size = 4) enrolled to receive neoadjuvant everolimus (10 mg/d, po) plus letrozole (2.5 mg/d, po) for 18 weeks or neoadjuvant FEC (fluorouracil 600 mg/m^2^, iv, d1 + epirubicin 90 mg/ m^2^, iv, d1 + cyclophosphamide 600 mg/m^2^, iv, d1; every 21 days per cycle) for 6 cycles before definitive surgery. In addition, prophylactic use of dexamethasone mouth wash could be used to reduce the everolimus-induced stomatitis in patients who receive neoadjuvant everolimus plus letrozole based on the result of SWISH trial [20]. Patient visits would be scheduled at 6, 12 and 18 weeks, and the preoperative time-point. At each visit medical history, physical examination, breast ultrasound, toxicity assessment, hematology and serum chemistry would be performed. Immune monitoring on peripheral blood CD4^+^ T cells, CD8^+^ T cells, tumor-specific cytotoxic lymphocytes (CTLs; IFN-γ-secreting CD8^+^ T cells activated by tumor lysis), Tregs, NK cells, CD4^+^ NKTs and CD8^+^ NKTs were performed at baseline and subsequently at 18 weeks (after the completion of neoadjuvant therapy). Lymph node surgery was performed at the same time as breast surgery. Sentinel lymph node biopsy was allowed only after completion of neoadjuvant therapy.

Adverse events were graded according to National Cancer Institute Common Toxicity Criteria (version 4.0). Treatment with everolimus would be interrupted for grade 1 thrombocytopenia that lasted greater than 2 weeks and for ≥grade 2 stomatitis or pneumonitis, any grade 3 hematologic or other non-hematologic toxicity. Treatment would resume with reduced dose of everolimus (5 mg/d) if recovery occurred. If toxicity persisted for more than 4 weeks or reappear after resolution, or if any grade 4 toxicity occurred, treatment with everolimus would be discontinued, and the patients would continue on letrozole until surgery.

If neutropenic fever or sepsis occurred after a cycle of chemotherapy, the next cycle would be delayed until the absolute neutrophil count is at least 1.0 × 10^9^ cells per L. After a delay, either dose reduction of all drugs to 80%, or granulocyte colony-stimulating factor (GCSF) support with 100% dose were allowed. For persistent thrombocytopenia, the next cycle would be delayed until platelets have recovered to at least 100 × 10^9^ cells per L, and chemotherapy doses would be reduced to 80%. Detailed procedures were described in our previous trial protocol paper [19].

### Outcomes

The primary endpoint was the feasibility of the trial. Secondary endpoints included ultrasound response rate (Objective response by ultrasound was defined by Response Evaluation Criteria in Solid Tumors (RECIST) version 1.1.), pCR rate, breast-conserving surgery rate, toxicities, and changes in the percentages of peripheral immune cell subsets, and changes of tumor Ki67 index, PD-L1 expression in tumor, and tumor-infiltrating CD8^+^ T cells, B cells and Tregs in relation to neoadjuvant therapy, as well as associations between these immune biomarkers and response to neoadjuvant therapy.

### Biomarker analysis

#### Flow cytometry

Peripheral blood from patients were collected before and after neoadjuvant therapy. For lymphocyte subsets detection, 50 μL peripheral blood were stained with FITC-conjugated anti-CD3, PE/Cy7-conjugated anti-CD4, APC/Cy7-conjugated anti-CD8, PE-conjugated anti-CD16/CD56, APC-conjugated anti-CD19, ECD-conjugated anti-CD20 and PerCP/Cy5.5-conjugated anti-CD45 for 30 min–45 min at 4 °C, respectively. For Tregs detection, whole blood were stained with FITC-conjugated CD4, PC5-conjugated CD25 and PE-conjugated CD127 for 45 min at 4 °C. After antibody staining, hemolysin was used to lyse RBC. Single cell suspension were washed and then resuspend with 200 μL staining buffer for flow cytometry. Flow cytometry was performed and analyzed on FACS Diva (BD bioscience). All antibodies were purchased from Beckman Coulter.

#### Immunohistochemistry

Tumor tissues excised from patients were fixed and embedded in paraffin, sectioned at 4 μm and stained with anti-CD8 (85,336 s, CST, 1:400), anti-PD-L1 (ab205921, Abcam; 1:100), anti-CD19 (ZM-0038; Zsbio; 1:100) and anti-FoxP3 (98,377 s, CST,1:200) at 4 °C overnight. Afterwards, biotin-conjugated secondary antibody, HRP-conjugated streptavidin and DAB kit were further performed to determine the positive signal. Images were obtained on a Nikon Ni-E microscope and analyzed using the Nikon NIS-Elements software package. Percentage of positive signal was counted in 5 randomized fields.

#### Induction of tumor-specific antigen loaded dendritic cells (DCs)

DCs were derived from cultured and induced peripheral blood mononuclear cell (PBMC) from patients. Briefly, PBMCs were collected by using lymphocytes separation medium (Ficoll), and cultured in DC-induction medium (RPMI-1640 containing 10% fetal bovine serum (Thermo Fisher Scientific), 20 ng/mL GM-CSF, 5 ng/mL IL-4 and 100 U/mL penicillin-streptomycin) for 7 days. At the time of day 7, PBMCs were induced into immature DCs. For DC maturation, immature DCs were first pulsed with tumor lysis (protein concentration: 200 μg/mL) for 3 h, after which pulsed DCs were cultured in maturation medium (containing 20 ng/mL TNFα) for 24 h. All cytokines were purchased from Peprotech. Mature tumor-specific antigen loaded DCs were ready for the antigen presentation.

#### Sortation of CD8^+^ T cells

15 mL peripheral blood was collected, and PBMCs were obtained by using lymphocytes separation medium (Ficoll). PBMCs were then stained with PerCP-conjugated CD3 and PE-conjugated CD8 for 30 min at 4 °C after which CD8^+^ T cells were sorted by FACS.

#### Detection of tumor-specific CTL

Tumor-specific CTL was detected by IFN-γ ELISOT (Dakewe). Briefly, sorted CD8^+^ T cells were co-cultured with autologous tumor-pulsed DCs (5:1) in anti-IFN-γ antibody pre-coated wells for 20 h at 37 °C. Afterwards, cells were lysed with H_2_O and incubated with biotin-conjugated anti-IFN-γ antibody and HRP-conjugated streptavidin orderly. Positive spot which represented a tumor-specific CTL was colored by AEC and counted.

### Statistical analysis

Because of the exploratory nature of this trial, statistical power was not calculated to assess specific study outcomes. The primary purpose of this study was to determine the feasibility (recruitment, completion of treatment per protocol, tissue collection, etc.) of the trial. Forty patients (20 per group) were recruited in this pilot study. This sample size calculation was developed in accordance with the recommendations by prior reports on sample size determination for pilot studies [21–23].

Statistical analyses were performed according to the intention-to-treat (ITT) principle. Patients who have taken their study medication on at least one occasion were considered evaluable cases. Ultrasound response rate, pCR rate, breast-conserving surgery rate, and toxicities were compared between the two arms using the chi-square test. Changes of peripheral and tumor biomarkers were analyzed via Wilcoxon matched-pairs signed rank test. Associations between peripheral and tumor biomarkers, and response to the neoadjuvant therapy were analyzed via chi-square test. Statistical analyses were conducted using STATA 12.0 software (StataCrop, College Station, TX). All statistical tests were two-sided, and statistical significance was defined as *P* < 0.05.

## Results

### Study patients

Between June 20th, 2016, and November 12th, 2019, 63 patients were screened. Among those, 40 patients were finally enrolled into the study and randomized (*n* = 20 in each arm) (Fig. 1). Eighteen patients and 14 patients completed the treatment as per protocol in the everolimus plus letrozole NET arm and the FEC NAC arm, respectively. Thus, the completion rate was 90.0% (95% CI, 68.3–98.8%) in the NET arm but 70.0% (95% CI, 45.7–88.1%) in the NAC arm. Key baseline characteristics of the patients in both cohorts are shown in Table 1. Demographic and clinicopathological features were well balanced between the two arms. The median age of diagnosis of was 60 (range 54–70) years old and 56.5 (range 51–66) in the everolimus plus letrozole NET arm and the FEC NAC arm, respectively. Five of Twenty (25%) patients in the everolimus plus letrozole NET arm had a tumor stage of T3/T4, while 4 of 20 (20%) patients in the FEC NAC arm had a higher tumor stage (T3/T4).

**Table 1:** Baseline characteristics

Characteristic, n (%)	Everolimus plus letrozole (***n*** = 20)	FEC (***n*** = 20)
**Age, year**		
Median (range)	60.0 (54.0–70.0)	56.5 (51.0–66.0)
**ER status**		
Positive	20 (100.0)	20 (100.0)
Negative	0 (0.0)	0 (0.0)
**PR status**		
Positive	19 (95.0)	14 (70.0)
Negative	1 (5.0)	6 (30.0)
**T stage**		
T1	2 (10.0)	1 (5.0)
T2	13 (65.0)	15 (75.0)
T3/T4	5 (25.0)	4 (20.0)
**N stage**		
N0	9 (45.0)	8 (40.0)
N1	11 (55.0)	12 (60.0)
**Histological type**		
Ductal	12 (60.0)	17 (85.0)
Lobular	3 (15.0)	0 (0.0)
Other/unknown	5 (25.0)	3 (15.0)
**Ki67 expression**		
< 20%	10 (50.0)	5 (25.0)
≥ 20%	10 (50.0)	15 (75.0)

Data were presented as median (range) or No. (%). *FEC* Fluorouracil, epirubicin plus cyclophosphamide, *ER* Estrogen receptor, *PR* Progesterone receptor

### Clinical activity

At final analysis, 13 of 20 patients in the NET arm achieved partial response (PR) before surgery, while 8 of 20 patients in the NAC arm achieved PR before surgery (Table 2). There was no patient had a complete response (CR) in each arm. The ultrasound response rate was 65.0% (95% CI 40.8–84.6) in NET arm and 40.0% (95% CI 19.1–63.9) in FEC arm, respectively. The pCR rate was 0% in each arm. In terms of the breast-conserving surgery rate, it was 45.0 and 25.0% in the NET arm and the NAC arm, respectively.

**Table 2:** Objective Response

Objective response (n, %)	Everolimus plus letrozole (***n*** = 20)	FEC (***n*** = 20)
Complete response	0 (0.0)	0 (0.0)
Partial response	13 (65.0)	8 (40.0)
Stable disease	6 (30.0)	10 (50.0)
Progressive disease	1 (5.0)	2 (10.0)

### Safety

In the NET group, the most common adverse events were stomatitis, and insomnia (Table 3). No cases of neutropenic infection or fever were observed. One (5.0%) of 20 patients had experienced grade 3–4 adverse events. The most common adverse events were nausea, and fatigue in the NAC arm (Table 3). 4 (20.0%) of 20 patients experienced grade 3–4 adverse events. Reasonably, patients in the NET group experienced significantly more stomatitis than those in the NAC group, whereas patients in the NAC group experienced significantly more leukopenia, neutropenia, nausea, decreased appetite, and alopecia than those in the NET group (Table 3). Serious adverse events (SAE) occurred in 1 patients (5.0%) in the NAC group, while there was no SAE in the NET group. A total of one patient (5.0%) had adverse events that led to discontinuation of chemotherapy, whereas there was no patient had any adverse events that led to discontinuation of everolimus plus letrozole. Three patients (15.0%) in the NET arm had adverse events led to a dose reduction or interruption of everolimus, while chemotherapy dose reduction or interruption were observed in 3 patients (15.0%) in the NAC arm.

**Table 3:** Treatment-related adverse events

System organ class preferred term, n (%)	Everolimus plus letrozole (***n*** = 20)		FEC (***n*** = 20)		***P*** value (all grades)
	All grades (%)	Grade 3–4 (%)	All grades (%)	Grade 3–4 (%)	
Mucositis oral	15 (75.0)	1 (5.0)	1 (5.0)	0 (0.0)	< 0.001
Anemia	10 (50.0)	0 (0.0)	10 (50.0)	0 (0.0)	1
Insomnia	6 (30.0)	0 (0.0)	1 (5.0)	0 (0.0)	0.092
Fatigue	4 (20.0)	0 (0.0)	11 (55.0)	0 (0.0)	0.048
Cough	4 (20.0)	0 (0.0)	3 (15.0)	0 (0.0)	1
Aspartate/Alanine aminotransferase increased	4 (20.0)	0 (0.0)	6 (30.0)	0 (0.0)	0.716
Bullous dermatitis/Rash maculo-papular	3 (15.0)	0 (0.0)	0 (0.0)	0 (0.0)	0.23
Oropharyngeal pain	3 (15.0)	0 (0.0)	0 (0.0)	0 (0.0)	0.231
Flu like symptoms	2 (10.0)	0 (0.0)	3 (15.0)	0 (0.0)	1
Nausea	1 (5.0)	0 (0.0)	13 (65.0)	0 (0.0)	< 0.001
Diarrhea	1 (5.0)	0 (0.0)	2 (10.0)	0 (0.0)	1
Constipation	1 (5.0)	0 (0.0)	3 (15.0)	0 (0.0)	0.605
Weight loss	1 (5.0)	0 (0.0)	0 (0.0)	0 (0.0)	1
Tumor pain	1 (5.0)	0 (0.0)	0 (0.0)	0 (0.0)	1
Hyperlipidemia	1 (5.0)	0 (0.0)	0 (0.0)	0 (0.0)	1
Pruritus	1 (5.0)	0 (0.0)	0 (0.0)	0 (0.0)	1
Toxic epidermal necrolysis	1 (5.0)	0 (0.0)	0 (0.0)	0 (0.0)	1
Bone pain	1 (5.0)	0 (0.0)	1 (5.0)	0 (0.0)	1
Non-cardiac chest pain	1 (5.0)	0 (0.0)	0 (0.0)	0 (0.0)	1
Paresthesia	1 (5.0)	0 (0.0)	0 (0.0)	0 (0.0)	1
Laryngeal inflammation	1 (5.0)	0 (0.0)	0 (0.0)	0 (0.0)	1
White blood cell decreased	1 (5.0)	0 (0.0)	9 (45.0)	4 (20.0)	0.008
Vomiting	0 (0.0)	0 (0.0)	1 (5.0)	0 (0.0)	1
Stomach pain	0 (0.0)	0 (0.0)	1 (5.0)	0 (0.0)	1
Anorexia	0 (0.0)	0 (0.0)	6 (30.0)	0 (0.0)	0.02
Fever	0 (0.0)	0 (0.0)	2 (10.0)	0 (0.0)	0.487
Alopecia	0 (0.0)	0 (0.0)	9 (45.0)	0 (0.0)	0.001
Generalized edema	0 (0.0)	0 (0.0)	1 (5.0)	0 (0.0)	1
Bone marrow hypocellular	0 (0.0)	0 (0.0)	1 (5.0)	0 (0.0)	1
Palpitations	0 (0.0)	0 (0.0)	1 (5.0)	0 (0.0)	1
Hot flashes	0 (0.0)	0 (0.0)	2 (10.0)	0 (0.0)	0.487
Headache	0 (0.0)	0 (0.0)	3 (15.0)	0 (0.0)	0.231
Neutrophil count decreased	0 (0.0)	0 (0.0)	6 (30.0)	4 (20.0)	0.02

Adverse events occurring in all enrolled patients were reported according to National Cancer Institute Common Toxicity Criteria (Version 4.0). Data were presented as number of patients (%). *FEC* Fluorouracil, epirubicin plus cyclophosphamide

### Biomarker analyses

All patients consented to collection of blood and tumor samples. Thirty-nine of Forty patients (97.5%) provided blood samples and 24 of those (60.0%) provided tumor samples at both time-points. Immunoregulatory effects of neoadjuvant therapy, and associations between blood immune cell subpopulations, tumor-specific CTLs as well as tumor Ki67 index and tumor immune biomarkers, and response to NET or NAC were assessed.

**Fig. 2:**
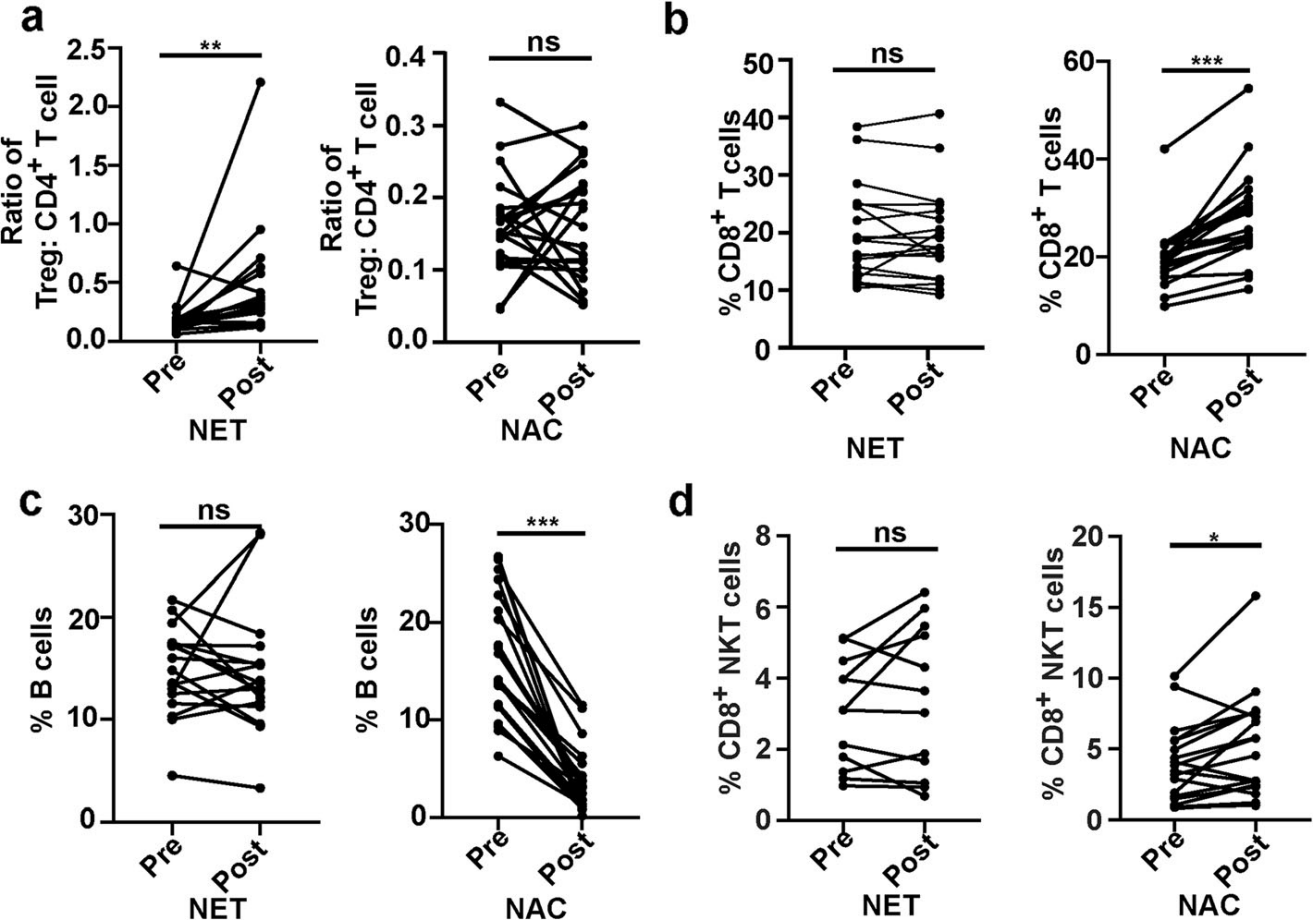
Neoadjuvant therapy-induced changes of blood lymphocyte subsets and ratio of Tregs to CD4^+^ T cells. PBMCs before neoadjuvant treatment (baseline) and after treatment were analyzed. **a**. Changes of ratio of Tregs to CD4^+^ T cells after neoadjuvant endocrine therapy (NET) and neoadjuvant chemotherapy (NAC). **b**. Changes of percentage of CD8^+^ T cells among PBMCs after NET and NAC. **c**. Changes of percentage of B cells among PBMCs after NET and NAC. **d.** Changes of percentage of CD8^+^ NKT cells among PBMCs after NET and NAC. Statistical analyses were performed by Wilcoxon matched-pairs signed rank test and *P* < 0.05 was considered statistically significant

Firstly, we aimed to verify the immunoregulatory effects of neoadjuvant therapy. We found that proportion of baseline immune cell subsets, as well as ratio of Tregs to CD4^+^ T cells and ratio of Tregs to CD8^+^ T cells showed no differences between NET and NAC group (Fig. S1a-j). Next, the changes of blood lymphocyte subsets after neoadjuvant therapy were assessed. In the NET group, no significant differences were observed in changes of immune cell subpopulations after therapy (Fig. S2a-f). However, we found that ratio of Tregs to CD4^+^ T cells increased significantly after NET (*P* = 0.002, Fig. 2a). In the NAC group, the population of CD8^+^ T cells significantly increased after chemotherapy (*P* < 0.001, Fig. 2b). Meanwhile, frequency of B cells markedly decreased after chemotherapy in the NAC group (*P* < 0.001, Fig. 2c). Furthermore, frequency of CD8^+^ NKT cells elevated after chemotherapy in the NAC group (*P =* 0.015, Fig. 2d).

In addition, we analyzed the number of circulating tumor-specific CTLs at baseline and post-therapy. IFN-γ ELISPOT assay showed that there were no differences of tumor-specific CTLs at baseline between two arms (*P* = 0.666, Fig. S3a). And neoadjuvant everolimus plus letrozole treatment or chemotherapy have not changed the number of tumor-specific CTLs (Fig. S3b-c).

**Fig. 3:**
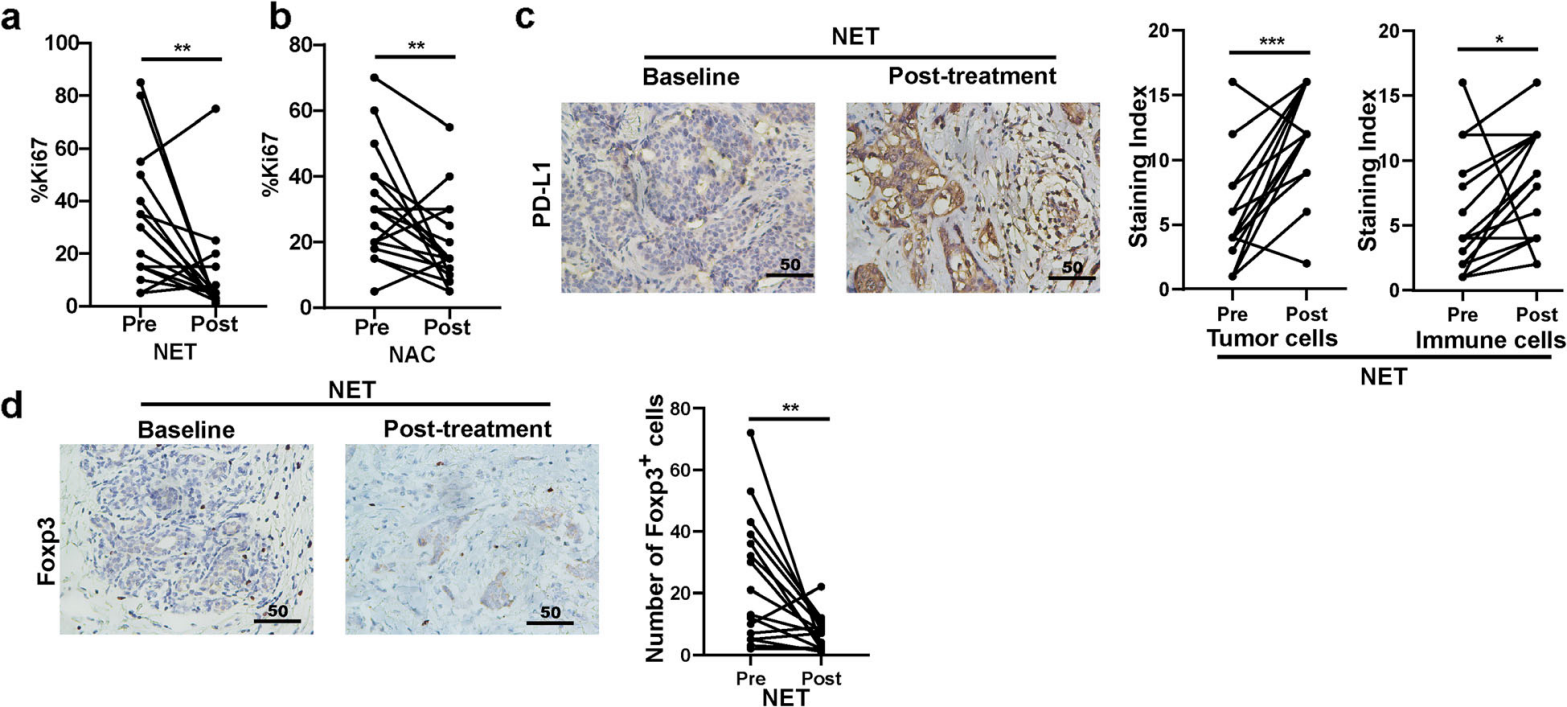
Neoadjuvant therapy-induced changes of biomarkers in tumor tissue. **a**. Changes of proportion of Ki67-positive tumor cells after neoadjuvant endocrine therapy (NET). **b**. Changes of proportion of Ki67-positive tumor cells after neoadjuvant chemotherapy (NAC). **c.** (left) Representative pictures of PD-L1 staining in biopsy samples (Baseline) and surgical samples (Post-treatment) from patients treated with NET. Scale bar: 400×. (right) Scatter plots showed the changes of PDL-1 staining index respectively in tumor cells and immune cells after NET. **d**. (left) Representative pictures of Foxp3 staining in biopsy samples (Baseline) and surgical samples (Post-treatment) from patients treated with NET. Scale bar: 400×. (right) A scatter plot showed the changes of number of Foxp3-positive cells in tumor tissues after NET. Statistical analyses were performed by Wilcoxon matched-pairs signed rank test and *P* < 0.05 was considered statistically significant

Moreover, we intended to explore the alteration of tumor biomarkers after neoadjuvant therapy. Baseline tumor Ki67 index showed no statistical differences between NET and NAC groups (*P* = 0.399, Fig. S4a). By analyzing alteration of Ki67 index after NET or NAC, we found that the Ki67 level significantly decreased after everolimus plus letrozole or chemotherapy (*P* = 0.007 and 0.008, respectively; Fig. 3a-b). In addition, we also studied the effects of NET and NAC on PD-L1 expression on tumor or immune cells, and tumor-infiltrating CD8^+^ T cells, B cells and Tregs. PD-L1 expression on tumor cells and immune cells significantly elevated after NET (*P* < 0.001 and *P* = 0.038, Fig. 3c), and tumor-infiltrating Tregs significantly decreased after NET (*P* = 0.004, Fig. 3d). However, there were no statistical changes of tumor-infiltrating CD8^+^ T cells and B cells after NET (*P* > 0.05 for all; Fig. S4b-c). Furthermore, no significant changes of these tumor immune biomarkers were observed after NAC (*P* > 0.05 for all).

**Fig. 4:**
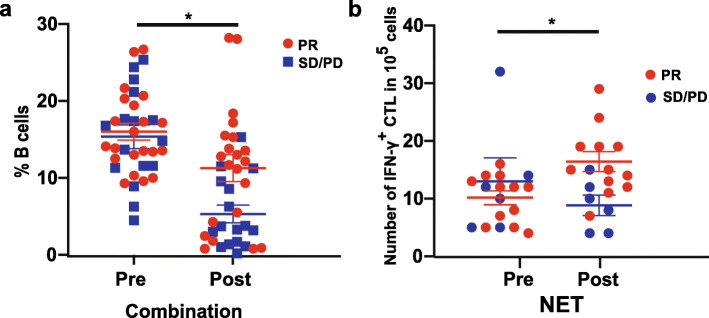
Associations between changes of blood immune cell subpopulations and therapeutic response after neoadjuvant therapy. **a**. Changes of percentage of B cells from responders (PR) or non-responders (SD/PD) after neoadjuvant therapy were analyzed. Non-responders (SD/PD) to neoadjuvant therapy had a greater decrease in blood B cell percentages compared to responders (PR) after neoadjuvant therapy. **b**. Changes of numbers of circulating tumor-specific CTLs (IFN-γ^+^ CTLs) from responders (PR) or non-responders (SD/PD) after NET were analyzed. Responders had a greater increase in the number of circulating tumor-specific CTLs compared to non-responders after NET. Statistical analysis was performed by Chi-square test and *P* < 0.05 was considered statistically significant

Next, the associations between blood immune cell subpopulations, tumor-specific CTLs, tumor Ki67 index and tumor immune biomarkers, and response to NET or NAC were assessed. There were no statistical differences between PR patients and SD/PD patients regarding proportion of circulating immune cell subsets at baseline or changes of proportions of immune cell subsets during therapies in both groups (Fig. S5A-G, Fig. S6a-f). However, combination analyses of patients from NET and NAC groups found that 81% of the SD/PD patients (13/16) showed a decrease > 35% in proportion of B cells, whereas only 43% of PR patients (9/21) had a decrease > 35% in proportion of B cells after neoadjuvant therapy (*P* = 0.018, Fig. 4a), indicating that non-responders to neoadjuvant therapy may had a greater decrease in B cell percentages compared to responders. We then evaluated the correlations between changes of blood tumor-specific CTLs during neoadjuvant therapy and clinical response. In the NET arm, 100% of PR patients (12/12) displayed an increased number of tumor-specific CTLs after neoadjuvant therapy, while only 17% of SD/PD patients (1/6) had an increased number after NET (*P* < 0.001; Fig. 4b), which suggested that responders may more likely to have tumor-specific CTLs increased after NET as compared with non-responders. Further, relationships between tumor Ki67 index and immune biomarkers, and response to treatment were analyzed. No statistical differences were found in baseline tumor Ki67 index between PR patients and SD/PD patients in the NET or NAC group (*P* = 0.544 and 1.000, respectively). Also, there were no statistical correlations between changes of tumor Ki67 index during therapy and the therapeutic response in neither NET nor NAC group (*P* = 1.000 and 0.425, respectively, Fig. S7). In addition, no significant correlations were found between baseline or changes of PD-L1 expression, tumor-infiltrating Tregs, CD8^+^ T cells and B cells, and response to NET or NAC therapy (*P* > 0.05 for all).

## Discussion

This is the first trial to determine the feasibility, efficacy and tolerability of head to head neoadjuvant everolimus plus letrozole versus neoadjuvant FEC chemotherapy in treating postmenopausal women with ER-positive, HER2-negative breast cancer. Moreover, this trial is the first study to provide valuable clinical data of the impact of everolimus plus letrozole which was applicated as new adjuvant therapy on peripheral blood immune cell subsets in postmenopausal ER-positive/HER2-negative patients. Here, we proved the feasibility of comparing everolimus plus letrozole NET with FEC NAC in postmenopausal ER-positive, HER2-negative breast cancer patients, and showed that the ultrasound response rate as well as the breast-conserving surgery rate was inspiring in everolimus plus letrozole arm, while the rates were relatively low in FEC chemotherapy arm. Results above provided the feasibility for further randomized controlled phase II or III trial with larger sample size. Additionally, we found that treatment of breast cancer patients with everolimus plus letrozole showed the tendency to increase the ratio of peripheral Tregs to CD4^+^ T cells and PD-L1 expression in tumor and decrease tumor Ki67 index and tumor-infiltrating Tregs, and patients with a greater increase of tumor-specific CTLs showed more sensitive to neoadjuvant everolimus in combination with letrozole. However, patients with a greater decrease in peripheral B cell percentages may benefit less from neoadjuvant therapies.

In this phase II pilot trial, the compliance rate of neoadjuvant everolimus plus letrozole (NET) was 90%, which was higher than that of FEC NAC arm (70%). The reason for lower compliance rate in NAC arm might be due to more severe adverse events and the situation that more patients suffered from progressive disease in NAC arm when compared to those in NET arm. Moreover, this trial demonstrated a favorable ultrasound response rate and breast-conserving surgery rate of neoadjuvant everolimus plus letrozole in treating postmenopausal women with ER-positive, HER2-negative breast cancer. Yet, pCR rate in both arms was 0%. It might due to the small sample size and relatively short treatment duration for application of neoadjuvant everolimus plus letrozole. In terms of the adverse events, clearly favored the everolimus plus letrozole endocrine therapy arm. These findings confirmed the potential efficacy and toxicity profile of neoadjuvant everolimus plus letrozole and of similar combinations in the advanced setting [24, 25]. However, it is different from the NeoPAL and PALLET trials, which both found very similar clinical response rates in patients underwent neoadjuvant CDK4/6 inhibitor palbociclib plus letrozole and neoadjuvant chemotherapies [10, 11]. Thus, everolimus might be an ideal drug combined with AIs that could sensitize breast tumors to neoadjuvant endocrine therapy. But the sample size of the current pilot study was small, a future large randomized controlled trial is needed to confirm our findings.

Previous preclinical and clinical studies reported that mTOR pathway has immunoregulation effects [15–18, 26]. However, no clinical studies have assessed the immunoregulatory effects of mTOR inhibitors in breast cancer patients so far. Here we found that neoadjuvant everolimus in combination with letrozole may increase the peripheral blood ratio of Tregs to CD4^+^ T cells. Our findings was consistent with a prior phase 2 trial of single-agent everolimus in chemotherapy-naive patients with castration-resistant prostate cancer, which also found that everolimus increased the percentage of blood Tregs [16]. However, we observed that neoadjuvant everolimus plus letrozole could decrease tumor-infiltrating Tregs, which has been reported as important cells that contribute to an immunosuppressive tumor microenvironment in breast cancer [27–29]. We also observed that neoadjuvant everolimus plus letrozole may increase the PD-L1 expression in tumor. A preclinical study found that PD-L1 expression was upregulated in tumor cells after everolimus treatment in renal cell carcinoma tumor-bearing mice, and the combination of everolimus with anti-PD-L1 antibody significantly reduced tumor burden compared with the everolimus alone, increasing tumor infiltrating lymphocytes (TILs) and the ratio of cytotoxic CD8^+^ T cells to TILs [30]. A phase Ib trial reported that first-line AKT inhibitor Ipatasertib in combination with atezolizumab and paclitaxel or nab-Paclitaxel in patients with advanced triple-negative breast cancer achieved a high ORR of 73% [31]. Together with these studies, our trial suggests that mTOR inhibitor everolimus might sensitize the checkpoint blockade including anti-PD-1 and anti-PD-L1 antibodies, which provides potential possibility for further studies focusing on anti-tumor activity of mTOR or AKT inhibitors combined with PD-1/PD-L1 blockade in treating HR+ and HER2- breast cancer patients.

Recent evidence suggests that tumor-infiltrating B cells may predict a better response to immunotherapy in several kinds of solid tumors, including melanoma, renal cell carcinoma, and sarcoma [32–34]. And our previous study investigated that advanced triple-negative breast cancer patients with an increase of tumor-infiltrating B cells after SHR-1210 (an anti-PD-1 antibody) plus apatinib (a VEGFR2 TKI) treatment benefited more from combinational therapy of immunotherapy and anti-angiogenic treatment [35]. However, very few studies have focused on peripheral B lymphocytes and response to anti-tumor treatment in breast cancer patients. In this study, we determined that patients with a greater decrease in peripheral B cell percentages may benefit less from neoadjuvant therapies, which suggests that circulating B cells may have an anti-tumoral immune activity in patients with HR+ and HER2- breast cancer. A significant decreased percentage of peripheral B cells after neoadjuvant therapy might identify a group of HR+ and HER2- breast cancer patients resistant to either NET or NAC. Also, we found that responders were more likely to have tumor-specific CTLs increased after NET as compared with non-responders, suggesting that changes of tumor-specific CTLs may involve in the anti-tumoral progression and predict the response to neoadjuvant everolimus plus letrozole therapy. This result showed a great potential to develop new anti-tumoral therapy targeting B cells or tumor-specific CTLs as well as new biomarkers for response to neoadjuvant therapy.

We are aware of the limitations of this study. In light of small sample size due to the pilot trial design, our conclusions must be interpreted with caution, and should be validated in future large randomized controlled trials. In addition, we enrolled limited luminal A breast cancer patients, which hinders the findings to luminal A subtype. Finally, the neoadjuvant chemotherapy arm in this study was 6 cycles of FEC, which is not a current standard NAC regimen. Therefore, in our future randomized controlled phase II or III trial with larger sample size, a NAC regimen including taxane (Fluorouracil, Epirubicin and Cyclophosphamide for 3 cycles followed by docetaxel for 3 cycles, or Epirubicin/Adriamycin combined with Cyclophosphamide for 4 cycles followed by docetaxel for 4 cycles) will be the neoadjuvant chemotherapy arm.

## Conclusions

This pilot trial demonstrated that neoadjuvant everolimus combined with letrozole might achieve a favorable ultrasound response rate with low toxicities in treating postmenopausal patients with ER-positive, HER2-negative breast cancer. Everolimus in combination with letrozole might have positive antitumoral immunity effects. Further large randomized controlled trials are required to confirm our findings.

## Supplementary Information

**Additional file 1: Figure S1.** Proportion of baseline blood lymphocyte subsets and the ratio of Tregs to CD4^+^ T, CD8^+^ T and NK cells in the neoadjuvant endocrine therapy (NET) and neoadjuvant chemotherapy (NAC) group. PBMCs before neoadjuvant treatment (baseline) were analyzed. Baseline percentages of CD4^+^ T cells (a), CD8^+^ T cells (b), Tregs (c), NK cells (d), B cells (e), CD4^+^ NKTs (f) and CD8^+^ NKTs (g), ratio of Tregs to CD4^+^ T cells (h), ratio of Tregs to CD8^+^ T cells (i) and ratio of Tregs to NK cells (j) in the NET and NAC group were shown as scatter plots. Statistical analyses were performed by Mann Whitney test and *P*<0.05 was considered statistically significant. **Figure S2.** Neoadjuvant therapy-induced changes of blood lymphocyte subsets, ratio of Treg to CD8^+^ T and ratio of Treg to NK cell. PBMCs before neoadjuvant treatment (baseline) and after treatment were analyzed. a. Changes of CD4^+^ T cell among PBMCs after neoadjuvant endocrine therapy (NET) or neoadjuvant chemotherapy (NAC). b. Changes of percentage of Tregs among PBMCs after NET or NAC. c. Changes of percentage of NK cells among PBMCs after NET or NAC. d. Changes of percentage of CD4^+^ NKT cells among PBMCs after NET or NAC. e. Changes of ratio of Tregs to CD8^+^ T cells after NET or NAC. f. Changes of ratio of Tregs to NK cells after NET or NAC. Statistical analyses were performed by Wilcoxon matched-pairs signed rank test and *P*<0.05 was considered statistically significant. **Figure S3.** Neoadjuvant therapy-induced changes of circulating tumor-specific CTLs. Blood samples at baseline and after neoadjuvant therapy were collected, and ELISPOT assays were performed to detect circulating tumor-specific CTLs (IFN-γ^+^ CTLs). Numbers of circulating tumor-specific CTLs before neoadjuvant treatment (baseline) and after treatment were analyzed. a. The number of baseline circulating tumor-specific CTLs (IFN-γ^+^ CTLs) between two groups were analyzed. b. Changes of circulating tumor-specific CTLs after neoadjuvant endocrine therapy (NET). c. Changes of circulating tumor-specific CTLs after neoadjuvant chemotherapy (NAC). Statistical analyses were performed by Mann Whitney test (a) or Wilcoxon matched-pairs signed rank test (b-c), and *P*<0.05 was considered statistically significant. **Figure S4.** Neoadjuvant therapy-induced changes of tumor biomarkers. a. Baseline proportions of Ki67-positive tumor cells in tumor samples in the neoadjuvant endocrine therapy (NET) and neoadjuvant chemotherapy (NAC) group. b. Changes of number of CD8^+^ cells in tumor tissues of patients after NET. C. Changes of number of CD19^+^ cells in tumor tissues of patients after NET. Statistical analyses were performed by Mann Whitney test (a) or Wilcoxon matched-pairs signed rank test (b-c), and *P*<0.05 was considered statistically significant. **Figure S5.** Associations between baseline proportions of blood lymphocyte subsets and therapeutic response in patients treated with neoadjuvant endocrine therapy (NET) or neoadjuvant chemotherapy (NAC). Baseline percentages of CD4^+^ T cells (a), CD8^+^ T cells (b), Tregs (c), NK cells (d), B cells (e), CD4^+^ NKTs (f) and CD8^+^ NKTs (g) from responders (PR) or non-responders (SD/PD) were analyzed. Statistical analyses were performed by Chi-square test and *P*<0.05 was considered statistically significant. **Figure S6.** Associations between changes of proportions of blood lymphocyte subsets and therapeutic response in patients treated with neoadjuvant endocrine therapy (NET) or neoadjuvant chemotherapy (NAC). Changes of percentages of CD4^+^ T cells (a), CD8^+^ T cells (b), Tregs (c), NK cells (d), CD4^+^ NKTs (e) and CD8^+^ NKTs (f) from responders (PR) or non-responders (SD/PD) were analyzed. Statistical analyses were performed by Chi-square test and *P*<0.05 was considered statistically significant. **Figure S7.** Associations between changes of proportions of Ki67-positive tumor cells in tumor tissues and therapeutic response in patients treated with neoadjuvant endocrine therapy (NET) or neoadjuvant chemotherapy (NAC). Changes of proportions of Ki67-positive tumor cells in tumor tissues in responders (PR) and non-responders (SD/PD) after (a) NET or (b) NAC were analyzed. Statistical analyses were performed by Chi-square test and *P*<0.05 was considered statistically significant.

## Data Availability

The datasets generated and/or analysed during the current study are available from the corresponding author on reasonable request.

## Abbreviations

NAC: Neoadjuvant chemotherapy
ER: Estrogen receptor
HER2: Human epidermal growth factor receptor 2
pCR: Pathological complete response
SEER: Surveillance, Epidemiology and End Results
NET: Neoadjuvant endocrine therapy
AIs: Aromatase inhibitors
FEC: Fluorouracil, epirubicin plus cyclophosphamide
Tregs: Regulatory T cells
NK: Natural killer
Ths: T helper cells
WBC: White blood cell
ALT: Alanine transaminase
AST: Aspartate transaminase
TBIL: Total bilirubin
CRF: Case report form
SAE: Serious adverse event
IDMB: Independent Data (and safety) Monitoring Board
ITT: Intention-to-treat
GCSF: Granulocyte colony-stimulating factor
