# Supplementary material for: Neoadjuvant everolimus plus letrozole versus fluorouracil, epirubicin and cyclophosphamide for ER-positive, HER2-negative breast cancer: a randomized pilot trial
Source: BMC Cancer. 2021 Jul 27;21:862. doi: 10.1186/s12885-021-08612-y (PMC8317384; doi:10.1186/s12885-021-08612-y)
Supplement: Supplementary file 1 — Additional file 1: Figure S1. Proportion of baseline blood lymphocyte subsets and the ratio of Tregs to CD4+ T, CD8+ T and NK cells in the neoadjuvant endocrine therapy (NET) and neoadjuvant chemotherapy (NAC) group. PBMCs before neoadjuvant treatment (baseline) were analyzed. Baseline percentages of CD4+ T cells (a), CD8+ T cells (b), Tregs (c), NK cells (d), B cells (e), CD4+ NKTs (f) and CD8+ NKTs (g), ratio of Tregs to CD4+ T cells (h), ratio of Tregs to CD8+ T cells (i) and ratio of Tregs to NK cells (j) in the NET and NAC group were shown as scatter plots. Statistical analyses were performed by Mann Whitney test and P<0.05 was considered statistically significant. Figure S2. Neoadjuvant therapy-induced changes of blood lymphocyte subsets, ratio of Treg to CD8+ T and ratio of Treg to NK cell. PBMCs before neoadjuvant treatment (baseline) and after treatment were analyzed. a. Changes of CD4+ T cell among PBMCs after neoadjuvant endocrine therapy (NET) or neoadjuvant chemotherapy (NAC). b. Changes of percentage of Tregs among PBMCs after NET or NAC. c. Changes of percentage of NK cells among PBMCs after NET or NAC. d. Changes of percentage of CD4+ NKT cells among PBMCs after NET or NAC. e. Changes of ratio of Tregs to CD8+ T cells after NET or NAC. f. Changes of ratio of Tregs to NK cells after NET or NAC. Statistical analyses were performed by Wilcoxon matched-pairs signed rank test and P<0.05 was considered statistically significant. Figure S3. Neoadjuvant therapy-induced changes of circulating tumor-specific CTLs. Blood samples at baseline and after neoadjuvant therapy were collected, and ELISPOT assays were performed to detect circulating tumor-specific CTLs (IFN-γ+ CTLs). Numbers of circulating tumor-specific CTLs before neoadjuvant treatment (baseline) and after treatment were analyzed. a. The number of baseline circulating tumor-specific CTLs (IFN-γ+ CTLs) between two groups were analyzed. b. Changes of circulating tumor-specific CTLs after neoadjuvant end [file 12885_2021_8612_MOESM1_ESM.docx]

Neoadjuvant Everolimus plus Letrozole Versus Fluorouracil, Epirubicin and Cyclophosphamide for ER-positive, HER2-negative Breast Cancer: a Randomized Pilot Trial

Wei Wu^1,3^, Jiewen Chen^1,3^, Heran Deng^1,3^, Liang Jin^1^, Zhanghai He^2^, Nanyan Rao^1^, Yan Nie^1^, Yandan Yao^1^, Yaping Yang^1^, Fengxi Su^1^, Jieqiong Liu^1,*^

^1^ Guangdong Provincial Key Laboratory of Malignant Tumor Epigenetics and Gene Regulation, Breast Tumor Center, Sun Yat-

sen Memorial Hospital, Sun Yat-sen University, Guangzhou, China.

^2^Guangdong Provincial Key Laboratory of Malignant Tumor Epigenetics and Gene Regulation, Department of Pathology, Sun Yat-sen Memorial Hospital, Sun Yat-sen University, Guangzhou, China.

^3^ Wei Wu, Jiewen Chen and Heran Deng contributed equally to this study.

^*^ Corresponding author: Jieqiong Liu, Guangdong Provincial Key Laboratory of Malignant Tumor Epigenetics and Gene Regulation, Breast Tumor Center, Sun Yat-sen Memorial Hospital, Sun Yat-sen University, Yanjiang West Road 107#, Guangzhou, China, 510120; Tel: 86-20-81332576; Fax: 86-20-34071156; E-mail: [liujieqiong01@163.com](mailto:liujieqiong01@163.com) or [liujq7@mail.sysu.edu.cn](mailto:liujq7@mail.sysu.edu.cn)

**Supplemental Materials**


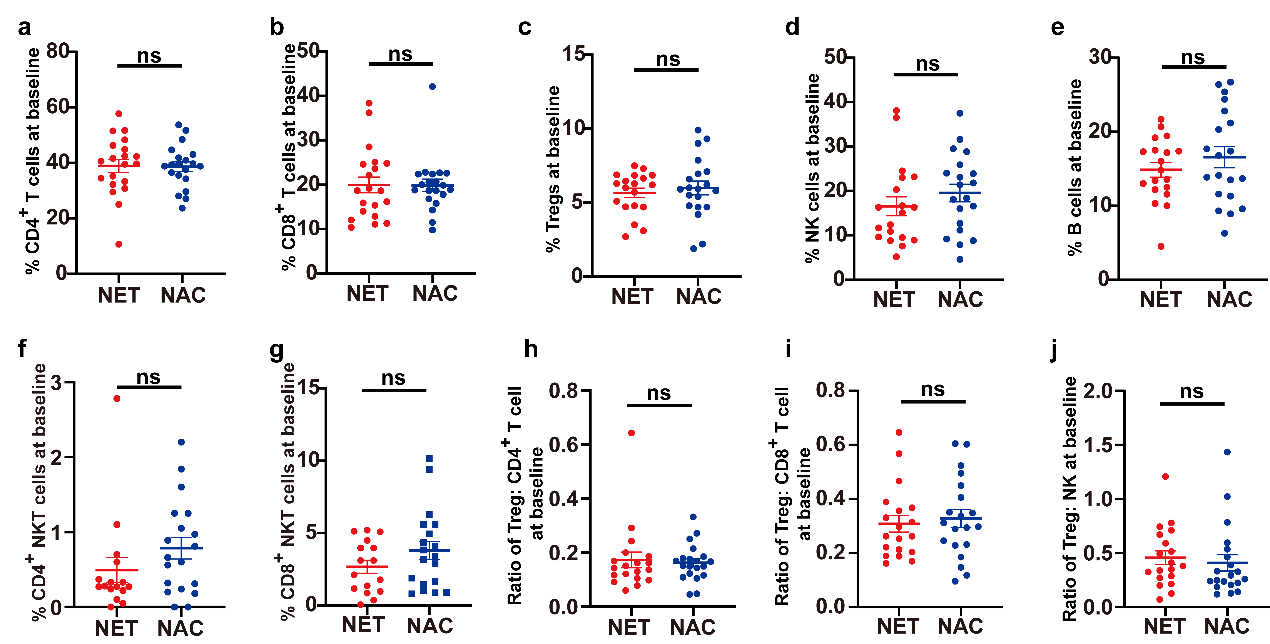


**Fig. S1** Proportion of baseline blood lymphocyte subsets and the ratio of Tregs to CD4^+^ T, CD8^+^ T and NK cells in the neoadjuvant endocrine therapy (NET) and neoadjuvant chemotherapy (NAC) group. PBMCs before neoadjuvant treatment (baseline) were analyzed. Baseline percentages of CD4^+^ T cells **(a)**, CD8^+^ T cells **(b)**, Tregs **(c)**, NK cells **(d)**,B cells **(e)**, CD4^+^ NKTs **(f)** and CD8^+^ NKTs **(g)**, ratio of Tregs to CD4^+^ T cells **(h)**, ratio of Tregs to CD8^+^ T cells **(i)** and ratio of Tregs to NK cells **(j)** in the NET and NAC group were shown as scatter plots. Statistical analyses were performed by Mann Whitney test and *P*<0.05 was considered statistically significant.


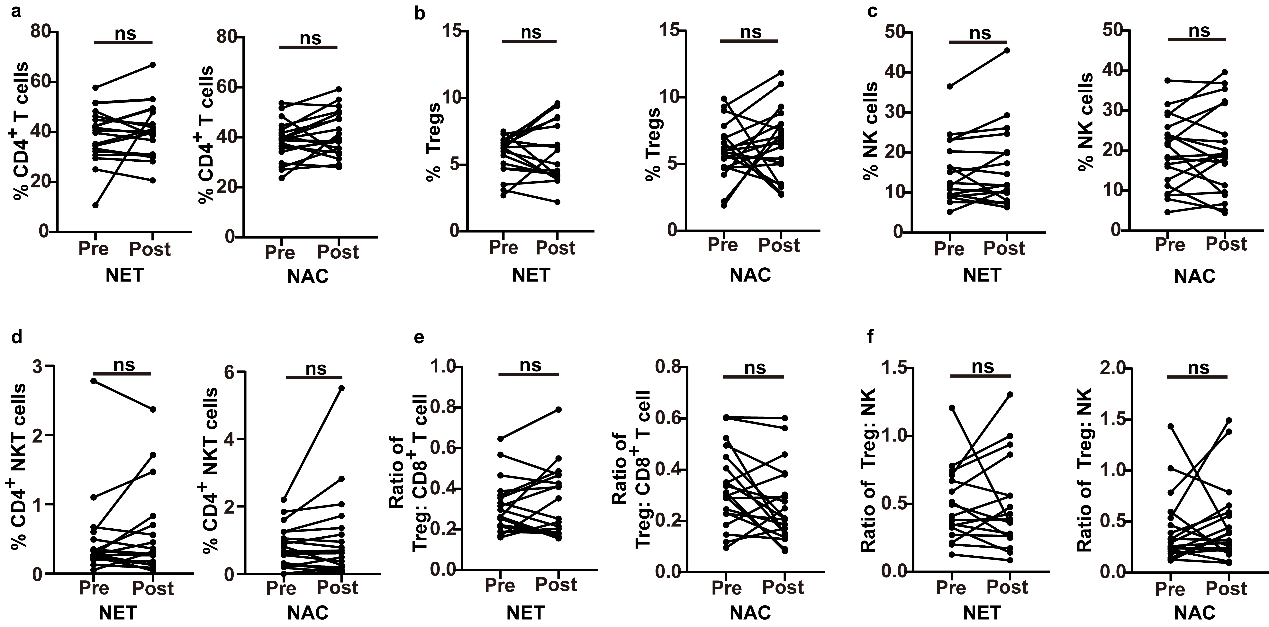


**Fig. S2** Neoadjuvant therapy-induced changes of blood lymphocyte subsets, ratio of Treg to CD8^+^ T and ratio of Treg to NK cell. PBMCs before neoadjuvant treatment (baseline) and after treatment were analyzed. **a.** Changes of CD4^+^ T cell among PBMCs after neoadjuvant endocrine therapy (NET) or neoadjuvant chemotherapy (NAC). **b.** Changes of percentage of Tregs among PBMCs after NET or NAC. **c.** Changes of percentage of NK cells among PBMCs after NET or NAC. **d.** Changes of percentage of CD4^+^ NKT cells among PBMCs after NET or NAC. **e.** Changes of ratio of Tregs to CD8^+^ T cells after NET or NAC. **f.** Changes of ratio of Tregs to NK cells after NET or NAC. Statistical analyses were performed by Wilcoxon matched-pairs signed rank test and *P*<0.05 was considered statistically significant.


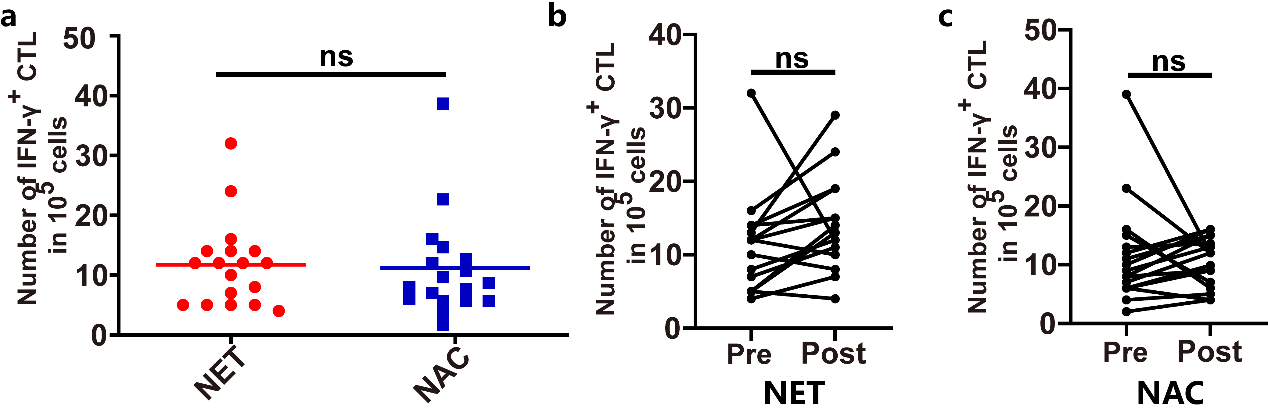


**Fig. S3** Neoadjuvant therapy-induced changes of circulating tumor-specific CTLs. Blood samples at baseline and after neoadjuvant therapy were collected, and ELISPOT assays were performed to detect circulating tumor-specific CTLs (IFN-γ^+^ CTLs). Numbers of circulating tumor-specific CTLs before neoadjuvant treatment (baseline) and after treatment were analyzed. **a.** The number of baseline circulating tumor-specific CTLs (IFN-γ^+^ CTLs) between two groups were analyzed. **b.** Changes of circulating tumor-specific CTLs after neoadjuvant endocrine therapy (NET). **c.** Changes of circulating tumor-specific CTLs after neoadjuvant chemotherapy (NAC). Statistical analyses were performed by Mann Whitney test (**a**) or Wilcoxon matched-pairs signed rank test (**b-c**), and *P*<0.05 was considered statistically significant.


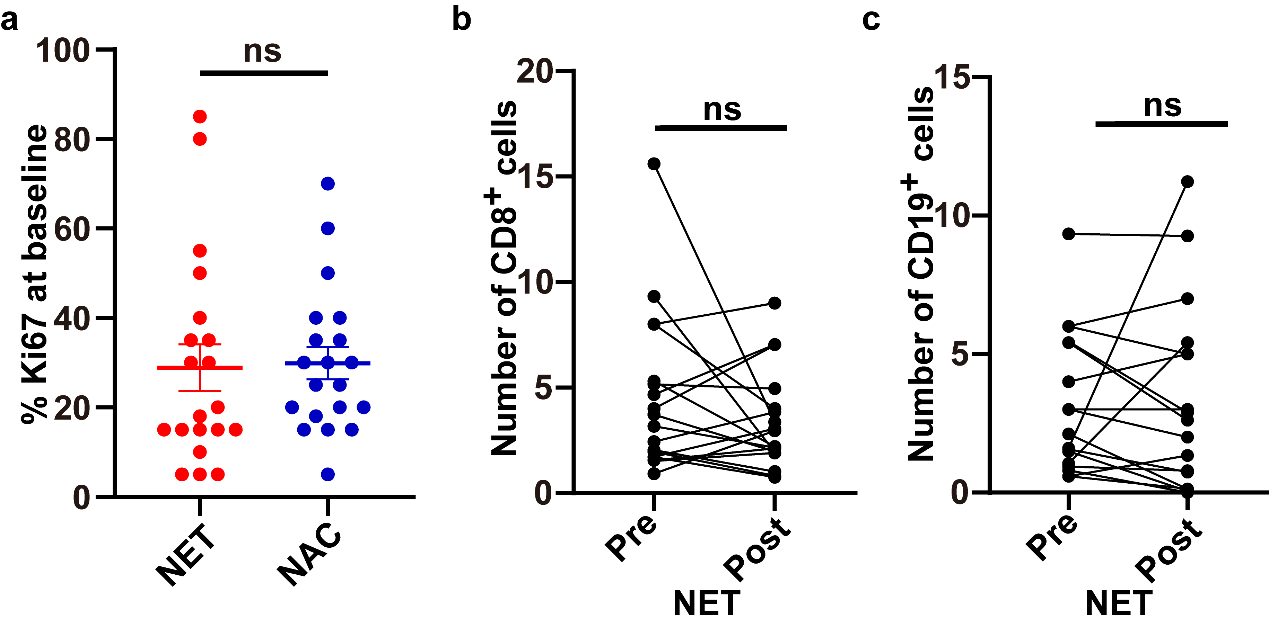


**Fig. S4** Neoadjuvant therapy-induced changes of tumor biomarkers. **a.** Baseline proportions of Ki67-positive tumor cells in tumor samples in the neoadjuvant endocrine therapy (NET) and neoadjuvant chemotherapy (NAC) group. **b.** Changes of number of CD8^+^ cells in tumor tissues of patients after NET. **C.** Changes of number of CD19^+^ cells in tumor tissues of patients after NET. Statistical analyses were performed by Mann Whitney test (**a**) or Wilcoxon matched-pairs signed rank test (**b-c**), and *P*<0.05 was considered statistically significant.


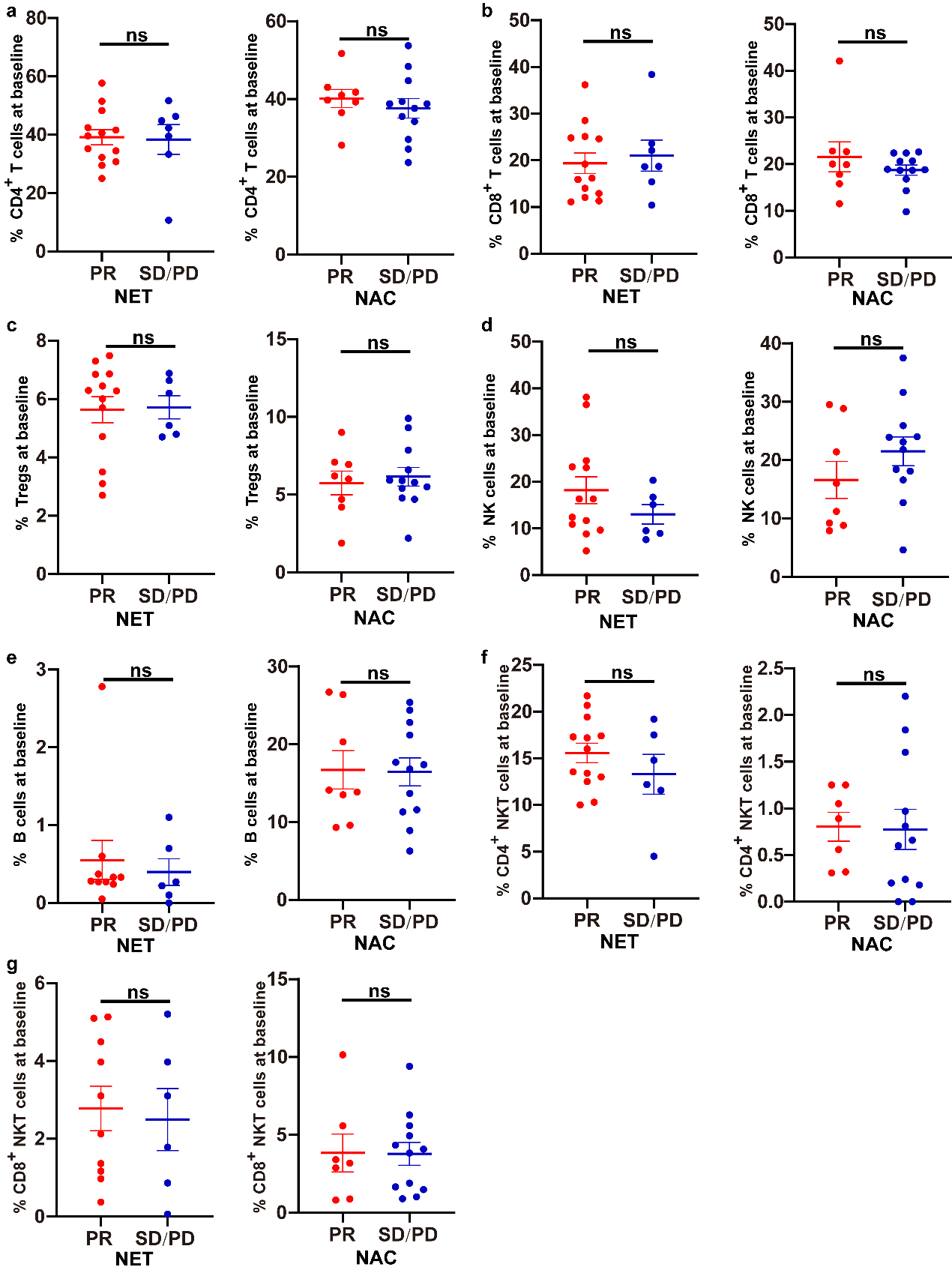


**Fig. S5** Associations between baseline proportions of blood lymphocyte subsets and therapeutic response in patients treated with neoadjuvant endocrine therapy (NET) or neoadjuvant chemotherapy (NAC). Baseline percentages of CD4^+^ T cells **(a)**, CD8^+^ T cells **(b)**, Tregs **(c)**, NK cells **(d)**, B cells **(e)**, CD4^+^ NKTs **(f)** and CD8^+^ NKTs **(g)** from responders (PR) or non-responders (SD/PD) were analyzed. Statistical analyses were performed by Chi-square test and *P*<0.05 was considered statistically significant.


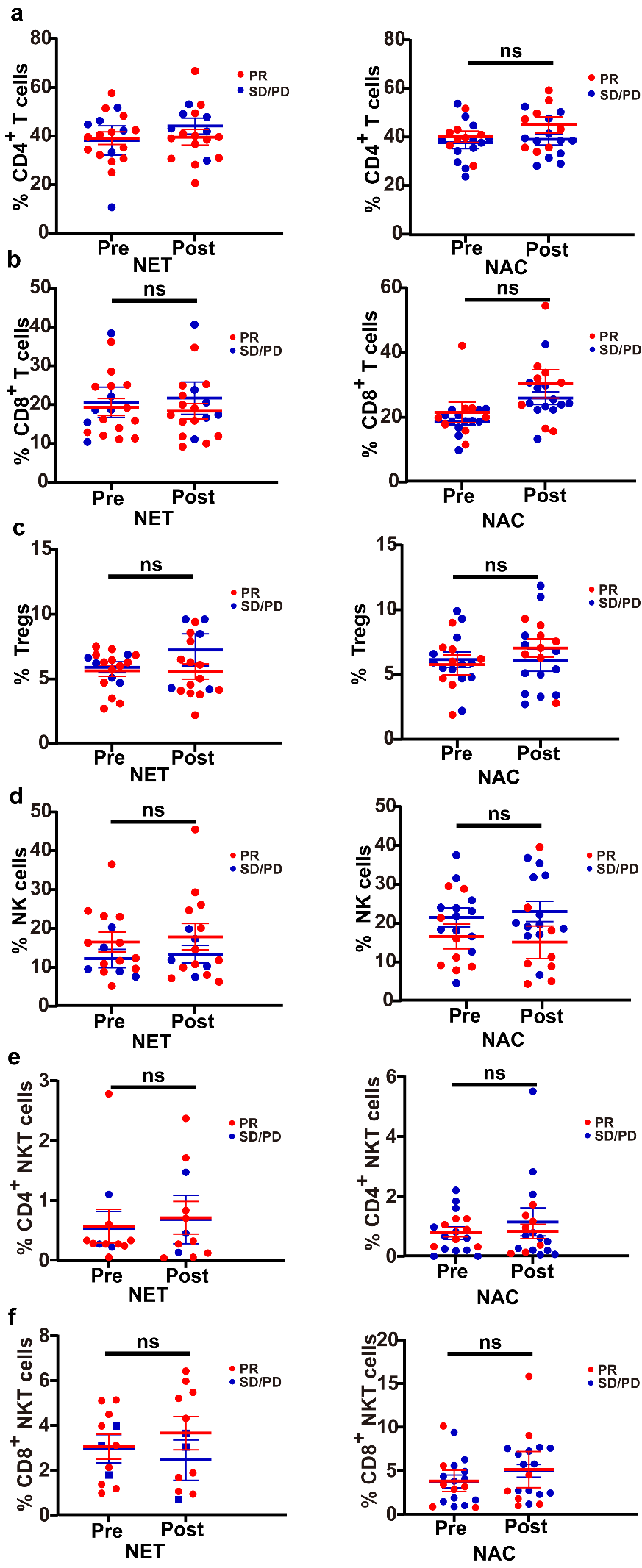


**Fig. S6** Associations between changes of proportions of blood lymphocyte subsets and therapeutic response in patients treated with neoadjuvant endocrine therapy (NET) or neoadjuvant chemotherapy (NAC). Changes of percentages of CD4^+^ T cells **(a)**, CD8^+^ T cells **(b)**, Tregs **(c)**, NK cells **(d)**, CD4^+^ NKTs **(e)** and CD8^+^ NKTs **(f)** from responders (PR) or non-responders (SD/PD) were analyzed. Statistical analyses were performed by Chi-square test and *P*<0.05 was considered statistically significant.


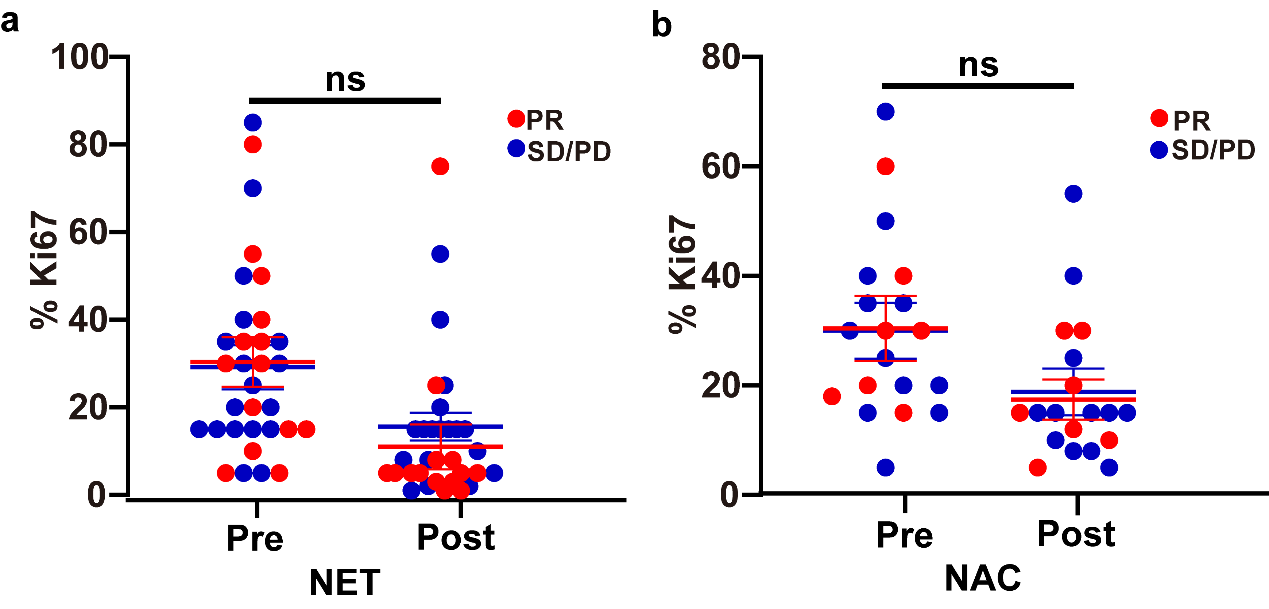


**Fig. S7** Associations between changes of proportions of Ki67-positive tumor cells in tumor tissues and therapeutic response in patients treated with neoadjuvant endocrine therapy (NET) or neoadjuvant chemotherapy (NAC). Changes of proportions of Ki67-positive tumor cells in tumor tissues in responders (PR) and non-responders (SD/PD) after **(a)** NET or **(b)** NAC were analyzed. Statistical analyses were performed by Chi-square test and *P*<0.05 was considered statistically significant.
